# Impact of Transitioning to Single-Patient Rooms on Prevention of Multidrug-Resistant Organisms in a Resource-Limited Facility

**DOI:** 10.1017/ash.2024.253

**Published:** 2024-09-16

**Authors:** Carol Fernandes, Camila Bezerra, Barbara Castro, Eusébio Santos Júnior, Maristela Freire, Susan Bollinger, Fernanda Lessa, Matias Salomao

**Affiliations:** Infection Control, Hospital das Clínicas, Universidade de São Paulo; Department of Infectious Diseases, Hospital das Clínicas, Universidade de São Paulo, São Paulo, Brazil; Universidade de São Paulo; Centers for Disease Control and Prevention; Hospital Das Clinicas Faculdade De Medicina Da Universidade De Sao Paulo

## Abstract

**Background:** Healthcare-associated infections (HAI) substantially increase hospital costs and lead to poor patient outcomes, particularly when caused by multidrug-resistant organisms (MDRO). To decrease MDRO transmission, isolation of colonized or infected patients in single rooms is recommended. However, single-patient isolation rooms are expensive to build and often unavailable in resource-limited hospitals. In 2023, an intensive care unit (ICU) at a large Brazilian tertiary hospital relocated from a space with an open floor plan to a newly built location with single-patient rooms. We evaluated the impact of this transition on acquisition of carbapenem-resistant Enterobacterales (CRE) colonization, HAI, and compliance with Hand Hygiene (HH) and Contact Precaution (CP) activities. **Methods:** We compared rates of CRE colonization acquisition, CRE colonization pressure, HAI, and compliance with HH and CP between pre- (March 1, 2022 – Feb 28, 2023) and post-implementation of single-rooms (March 1, 2023 - October 31, 2023) in a 12-bed surgical ICU. All patients were screened for CRE colonization on admission to the unit and weekly until discharge using rectal swab cultures. Colonization pressure was defined as the ratio of CRE-positive patient-days (PDs) to the total number of PDs. Rates of central-line associated blood-stream infections, ventilator-associated pneumonia, and catheter-associated urinary tract infections were monitored. HH and CP compliance were monitored weekly by infection prevention staff outside of the unit. Poisson regression and multiple linear regression were used to compare rates between pre- and post-implementation periods. **Results:** Healthcare acquisition of CRE colonization remainedstable between pre- and post-implementation (incidence rate ratio: 0.88 (95%CI, 0.73-1.05; P=0.16) despite an increase in CRE colonization pressure of 8.6% over baseline (from 7.84% pre- to 16.39% post-implementation (95% confidence interval [CI], 4.13-12.96%; P=.001)). The latter was driven by reduced turnover of CRE-colonized patients in the post-implementation period (mean patient-day reduced by 10.33; 95%CI, 3.06-17.61; P=0.006). Incidence of HAIs also remained stable (global incidence 3.12 vs 3.30, pre- and post-intervention, respectively; P=0.2). HH compliance was high prior to the transition (95.7%) and increased slightly but not significantly post-intervention (97.5%; P=0.3). CP compliance improved by 9.83%, especially in gown and glove changes after each patient interaction, from 90.62% pre- to 100% post-implementation (95%CI, 1.52-17.22; P=.02). Conclusion The move to an ICU with exclusively single-patient rooms was associated with increase in CP compliance. This could help explain why HAI incidence and healthcare acquisition of CRE colonization remained stable despite a significant increase in CRE colonization pressure.

**Disclosure:** Matias Salomao: Speaker - Cepheid

